# ECS-NL: An Enhanced Cuckoo Search Algorithm for Node Localisation in Wireless Sensor Networks

**DOI:** 10.3390/s21113576

**Published:** 2021-05-21

**Authors:** Vaibhav Kotiyal, Abhilash Singh, Sandeep Sharma, Jaiprakash Nagar, Cheng-Chi Lee

**Affiliations:** 1 Department of Industrial and Management Engineering, Indian Institute of Technology Kanpur, Kanpur 208016, India; kotiyalvaibhav98@gmail.com; 2 Fluvial Geomorphology and Remote Sensing Laboratory, Indian Institute of Science Education and Research Bhopal, Bhopal 462066, India; sabhilash@iiserb.ac.in; 3 Madhav Institute of Technology and Science, Gwalior 474005, India; 4 Indian Institute of Technology Kharagpur, Subir Chowdhury School of Quality and Reliability, Kharagpur 721302, India; jpnagar91@gmail.com; 5 Department of Library and Information Science, Research and Development, Center for Physical Education, Health, and Information Technology, Fu Jen Catholic University, New Taipei 242, Taiwan; 6 Department of Photonics and Communication Engineering and Department of Computer Science and Information Engineering, Asia University, Taichung 413, Taiwan

**Keywords:** WSNs, node localisation, bio-inspired algorithms, Cuckoo Search, ECS, optimisation function

## Abstract

Node localisation plays a critical role in setting up Wireless Sensor Networks (WSNs). A sensor in WSNs senses, processes and transmits the sensed information simultaneously. Along with the sensed information, it is crucial to have the positional information associated with the information source. A promising method to localise these randomly deployed sensors is to use bio-inspired meta-heuristic algorithms. In this way, a node localisation problem is converted to an optimisation problem. Afterwards, the optimisation problem is solved for an optimal solution by minimising the errors. Various bio-inspired algorithms, including the conventional Cuckoo Search (CS) and modified CS algorithm, have already been explored. However, these algorithms demand a predetermined number of iterations to reach the optimal solution, even when not required. In this way, they unnecessarily exploit the limited resources of the sensors resulting in a slow search process. This paper proposes an Enhanced Cuckoo Search (ECS) algorithm to minimise the Average Localisation Error (ALE) and the time taken to localise an unknown node. In this algorithm, we have implemented an Early Stopping (ES) mechanism, which improves the search process significantly by exiting the search loop whenever the optimal solution is reached. Further, we have evaluated the ECS algorithm and compared it with the modified CS algorithm. While doing so, note that the proposed algorithm localised all the localisable nodes in the network with an ALE of 0.5–0.8 m. In addition, the proposed algorithm also shows an 80% decrease in the average time taken to localise all the localisable nodes. Consequently, the performance of the proposed ECS algorithm makes it desirable to implement in practical scenarios for node localisation.

## 1. Introduction

Wireless sensor networks are made of a finite number of spatially distributed sensors and are self-configurable, requiring no pre-installed infrastructure for their effective operations. A wireless sensor network (WSN) can be installed on-the-fly in almost no time; as a result, they have applications in nearly every walk of life, such as in health, environmental monitoring, infrastructure monitoring, telecommunication, Internet of Things (IoT), precision agriculture, intrusion detection, disaster management, military surveillance, reconnaissance and so on [1,2,3,4,5,6,7,8,9,10]. The basic building blocks of these networks are sensors. Typically, each sensor has a sensing unit, power unit, processing unit and an antenna to receive or transmit the sensed information. Sensors are inherently resource-constrained as they have a limited battery, limited storage capacity, and limited processing power due to their compact size [11,12].

WSNs possess considerable potential for monitoring large or complicated-to-reach areas such as forest fire monitoring [13,14]. After the initial deployment, the controller needs to be aware about the locations being monitored. Therefore, it becomes mandatory to have information about the physical positions of the sensor nodes to gain critical information about the events occurring inside the region of interest. Any uncertainty in the positional information would result in an insignificant or erroneous analysis of the data gathered by the sensor nodes. At this point, we can utilise an effective localisation algorithm that can exploit the available nodes’ location information to localise the remaining nodes of the network. In this way, we can reduce the hardware on each sensor node, which would reduce the entire network’s cost. The localisation algorithms aim to estimate the accurate positions of the unknown nodes at the earliest [15].

Due to limited sensor life and losses involved in the communication of sensed information, it is challenging to develop a single localisation algorithm for all the possible applications of WSNs. Therefore, a trade-off must be made between the localisation error and the localisation time to achieve the optimal localisation results.

Different conventional approaches for node localisation have been discussed thoroughly in the literature [16,17,18,19]. These localisation algorithms are majorly divided into two phases: distance measurement phase using Residual Signal Strength Indication (RSSI), Angle of Arrival (AoA), Time of Arrival (ToA), etc. and position calculation phase using triangulation and trilateration. Niculescu et al. [20] proposed an Ad hoc Positioning System (APS), which increases the potential of the limited number of Global Positioning System (GPS) powered anchor nodes to all other non-GPS nodes in a hop-by-hop manner. In this method, first, the anchor nodes flood their positional information to all other nodes. Then, the unknown nodes use the GPS triangulation method to estimate their respective locations. Although this method is quite efficient, the communication overheads are significant due to regular flooding of positional information by the anchor nodes, affecting the network’s lifetime adversely. The communication overhead problem also occurs when the nodes keep moving, and the anchor nodes re-flood their positional information to update the network. Signal strength measurement errors are also observed in this process. To overcome the problem of communication overheads, an algorithm based on Multidimensional Scaling named MDS-MAP is proposed in [21]. It is a localisation method based on multidimensional scaling that uses the network nodes’ connectivity information to derive the locations of the unknown nodes. The application scope of MDS-MAP was reduced due to its centralised nature. To overcome this issue, Shang et al. [22] proposed a new version of MDS-MAP called MDS-MAP(P). This is a distributive method which generates maps or relative positions of neighbouring nodes. It also involves creating a local map for the immediate vicinity of each node and then merging all the maps to form a global map. This algorithm is best suited for near-uniform radio propagation, whereas radio communications are not practically uniform [23]. With the increase in the density of sensor nodes, the relative maps count will also increase, which would demand more storage capacity and battery consumption from the resource-limited sensors. Therefore, the use of this approach for the best possible solution demands a high computational cost. A robust and scalable method of formulating noise effects on signals using distributed processing techniques was proposed by Biswas et al. [24]. In this approach, the node localisation problem was solved with the help of an Semi-Definite Programming (SDP)-based optimisation technique having incomplete distance information. SDPs involve solving mathematical equations which increase the computational effort of resource-constrained sensor nodes.

All the conventional methods discussed above work reasonably well for the localisation of sensor nodes. However, they require extensive computation power, which increases with the increase in computational complexity. To minimise the computational cost, researchers have used bio-inspired meta-heuristic algorithms due to their capability to find optimal or near-optimal solutions in a reasonable time. They are known to consume less memory and computational time. Blum et al. [25] surveyed the most critical meta-heuristics from a conceptual point of view. These bio-inspired meta-heuristic algorithms are inspired from nature and considered some of the most efficient tools to solve various optimisation problem [11]. The task of node Localisation in WSN is considered as an unconstrained optimisation problem [26]. Several studies have [27,28,29] analysed and compared the performances of the bio-inspired algorithms with respect to the node localisation problem. Gopakumar et al. [30] proposed a WSNs localisation scheme based on Particle Swarm Optimisation (PSO) [31]. PSO is an evolutionary computation technique that simulates the social behaviour of bird flocking. This computationally efficient method is easy to implement [32,33,34]. The proposed localisation method assumes a centralised architecture for the WSNs, so that all the neighbouring anchor nodes can communicate to a central entity where the PSO can be implemented. However, this method is very prone to becoming stuck in a local optimum resulting in pre-mature convergence. An optimisation algorithm called Flower Pollination (FP) algorithm was proposed by Goyal et al. [35]. This algorithm is inspired by the pollination behaviour of the flowers. The basic idea is to estimate the best positions of the unknown nodes by moving the particles closer to optimum in every iteration. This algorithm was first tested in a small area with high anchor node density, which was not enough to prove this approach to be scalable. Furthermore, the complexity of this algorithm was due to the involvement of several rules. Goyal et al. [36] proposed a node localisation scheme-based CS algorithm. This approach converged to the optimum value slowly due to the random walk step size and the mutation probability being constants. In another work, Cheng et al. [37] modified the standard CS algorithm [38] by modifying the random walk step size and mutation probability to employ global search efficiently. Meta-heuristic algorithms select the best solution from a group of candidate solutions. After every iteration, the worst solutions out of the set of candidate solutions are replaced by new solutions. The probability of a bad solution to be replaced is called mutation probability. The approach used by Cheng et al. increased the convergence rate of the problem. The performance showed better results than leading meta-heuristic algorithms such as CS, PSO, Simulated Annealing (SA) and Genetic Algorithm (GA). Even after a fast convergence rate, the algorithm tends to run iterations up to the upper limit, and thus exploits the resources of the resource-constrained sensor nodes.

In this paper, we have proposed an Enhanced Cuckoo Search (ECS) algorithm to overcome the resource exploitation problem associated with the standard CS and the modified CS algorithms. In the proposed algorithm, the search process is not required to iterate for the predefined upper iteration limit to find the optimal solution in the search space. We have implemented an ES mechanism to exit the search loop whenever the search reaches the optimal value. This way, the computational overhead and the time taken to localise the localisable nodes reduce significantly.

Further, we have divided this paper into four sections: In Section 2, we have discussed the system model for the node localisation problem. Afterwards, in Section 3, we have discussed the simulation parameters and setup based on which the proposed ECS algorithm is evaluated. Furthermore, this section provides the details of the other simulation parameters used in the ECS algorithm. In  Section 4, we have discussed the simulated results and evaluated the potential of the proposed algorithm. Finally, in Section 5 and Section 6 we presented the discussion and conclusion, respectively.

## 2. System Model

In this section, we discuss the proposed system model to solve the node localisation problem. The system model incorporates an overview of the system architecture, distance calculation method and operational scenarios. Further, we render a detailed description of the proposed ECS algorithm’s objective function formulation and working. Finally, we discuss how ECS is executed to solve the node localisation problem.

### 2.1. System Architecture

**Figure 1:**
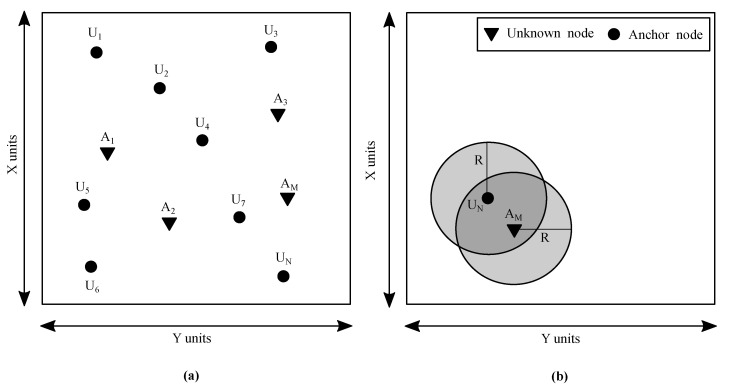
(**a**) System architecture with *M* anchor (in black-filled circle) and *N* unknown nodes (in black-filled triangle) deployed in X×Y square units area. (**b**) Sensor nodes with transmission range *R* distance units.

This study considers a rectangular-shaped monitoring region of dimensions X×Y and area *A* in square units (see Figure 1). A set of (M+N) sensor nodes is deployed randomly over the entire monitoring area. Out of all these deployed nodes, *M* nodes are aware of their position and are called anchor nodes. The remaining *N* nodes are unknown, and their positions are determined using the node localisation process. Each sensor node is assumed to have a homogeneous transmission range represented by *R*.

Each node in the model can transmit/receive data over a transmission range of *R* distance units. The anchor nodes broadcast their location information messages all over their transmission ranges along with their node IDs. It is practical to have anchor nodes broadcast their node IDs to all the neighbourhood nodes so that the unknown nodes recognise them. All the anchor nodes acquire their node IDs at the time of deployment. On the other hand, the unknown nodes receive the broadcast messages from the anchor nodes and calculate the received power of the message signal to compute the distance between them.

### 2.2. Distance Calculation Model

As discussed in Section 2.1, the unknown nodes calculate their distances from the anchor nodes with the help of received anchor message signal power. This power calculation is carried out with the help of RSSI. The RSSI indicates how well the receiver has received the signal sent by the transmitter. It is preferred because its measurement does not require external hardware and is easy to implement. Although this method does not provide accurate distance measurements, the measurements are good enough. The distance between two sensor nodes is calculated by using the path loss model [39,40] given by Equation (1):

(1) $$PL\left(d\right)\;=\;PL_{0}+10\eta \log_{10}\left(\frac{d}{d_{0}}\right)+X_{g}$$

where PL(d) represents the total path loss (transmitted power–received power); $PL_{0}$ represents the path loss with respect to a reference distance, $d_{0}$; *d* represents the distance between the transmitter and the receiver; and η represents the path loss exponent, indicating the rate at which the received signal strength decreases concerning distance; its value depends upon the propagation environment. $X_{g}$ is a Gaussian random variable representing the attenuation caused by fading. The ranging error is the result of log-normal shadowing with a zero-mean Gaussian distribution whose variance ($\sigma^{2}$) is given by

(2) $$\sigma^{2}\;=\;\gamma^{2}\times D_{ij}^{2}$$

where γ is the noise factor (γ = 0.1 in our study) and $D_{ij}$ is the Euclidean distance between the *i*-th node $\left(x_{i},y_{i}\right)$ and the *j*-th node $\left(x_{j},y_{j}\right)$ within the communication range. Equation (2) shows that the standard deviation of the ranging error is directly proportional to the actual distance between the nodes. The actual distance $D_{ij}$ can be calculated using

(3) $$D_{ij}\;=\;\sqrt{{\left(x_{i}-x_{j}\right)}^{2}+{\left(y_{i}-y_{j}\right)}^{2}}$$

A simple binary disk model has been adopted to establish network connectivity, i.e., two nodes *i* and *j* can communicate with each other iff $D_{ij}\leq R$, where *R* is the transmission range of sensor nodes. The measured distance is represented by $D_{ij}^{\prime }$ and is given by

(4) $$D_{ij}^{\prime }\;=\;D_{ij}+N_{ij}$$

where $N_{ij}$ represents the ranging error between the *i*-th and the *j*-th node locations.

### 2.3. Operation Scenarios

There exist two practically possible operating scenarios, i.e., most likely scenario and diverse scenario.

**Figure 2:**
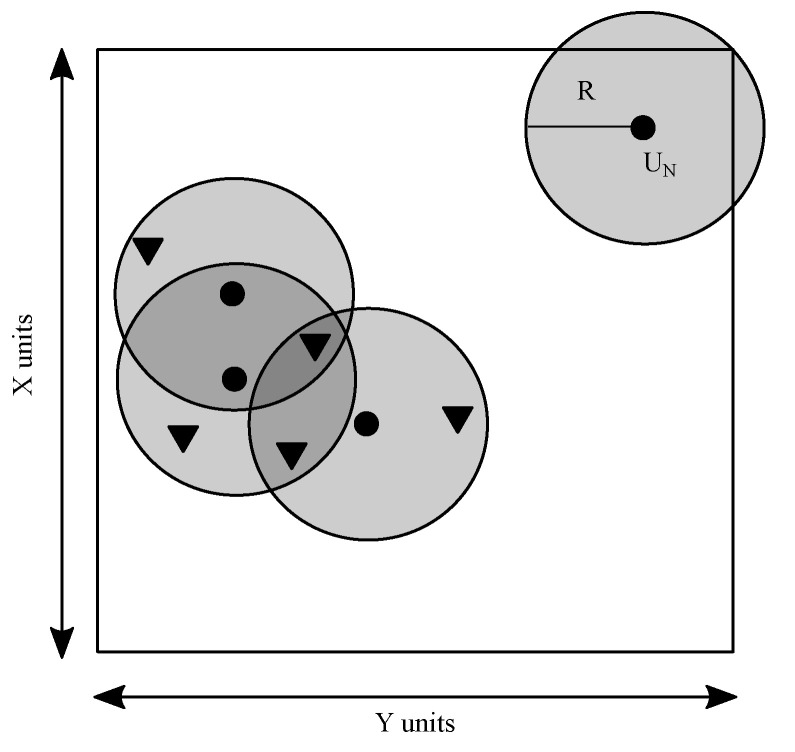
Diverse scenario in wireless sensor networks (WSNs) deployment.

(i) Most Likely Scenario
  This scenario is most likely to happen during the testing phase. Here, *M* number of anchor nodes and *N* number of unknown nodes would be placed randomly and each unknown node would call the ECS algorithm to localise itself. In this way, all the nodes are likely to localise themselves using the ECS node localisation process. The ranging error between any two arbitrary nodes depends on the Euclidean distance between them and is independent of the ranging error between any other two nodes of the network.
(ii) Diverse Scenario
  This scenario is infrequent to be implemented in a network, yet essential to consider. Any unknown node requires at least three or more anchor nodes within its transmission range to become localised. This scenario occurs when an unknown node gets deployed in a deserted area with no minimum anchor nodes around it, which prohibits the unknown node from getting localised. Figure 2 illustrates the diverse scenario of operation, which is also the limitation scenario of node deployment and is outside the control domain of node localisation. Consequently, the unknown nodes would remain unlocalised.

### 2.4. Optimisation Problem Formulation

Our experiment aims to estimate the position of an unknown node accurately. There will always exist some ranging error between the two nodes’ locations. The focus of this subsection is to formulate an objective function that minimises this ranging error.

Thus, an objective function (Obj) is established, which is the Mean Square Error (MSE) between the actual distance of estimated node coordinates and the estimated distance of actual unknown node coordinates from the neighbouring anchor nodes. Let $\left(x_{i},y_{i}\right)$ be the position of the *i*-th unknown node and $\left(x_{j},y_{j}\right)$ be the position of the *j*-th anchor node. The Obj is given by Equation (5):

(5) $$Obj{\left(x_{i},y_{i}\right)}_{min}\;=\;\frac{1}{M}\times \sum_{j=1}^{M}{\left(D_{ij}-D_{ij}^{\prime }\right)}^{2}$$

where M≥3, because an unknown node should have at least three anchor nodes within its transmission range to be considered localisable (trilateration rule). The $\left(x_{i},y_{i}\right)$ corresponding to the minimum value of the Obj is the estimated position of the unknown node.

### 2.5. ECS Algorithm

The ES criterion is built by making a record of the optimal values of the last few iterations. Let us assume, the optimum search values for iterations n,(n+1),(n+2) and (n+3) are $V_{n}$, $V_{n+1}$, $V_{n+2}$ and $V_{n+3}$ respectively. Then, the implementation of ES criterion is demonstrated as

(6) $$ES=\left\{\begin{array}{cc} True, & \Delta_{1}=\Delta_{2}=\Delta_{3}=0\\ False, & otherwise \end{array}\right.$$

where,

$$\begin{array}{ccc} \Delta_{1} & = & V_{n+1}-V_{n}\\ \Delta_{2} & = & V_{n+2}-V_{n+1}\\ \Delta_{3} & = & V_{n+3}-V_{n+2} \end{array}$$

This check is applied at the end of each iteration, and the search loop is exited when the ES criterion is satisfied. Implementation of the ES criterion shows up promising results, as it saves a lot of computational time and resources for localisation of the unknown nodes in the network. Algorithm 1 shows the proposed ECS algorithm for node localisation in WSNs.

### 2.6. Proposed Node Localisation Process in WSNs

**Figure 3:**
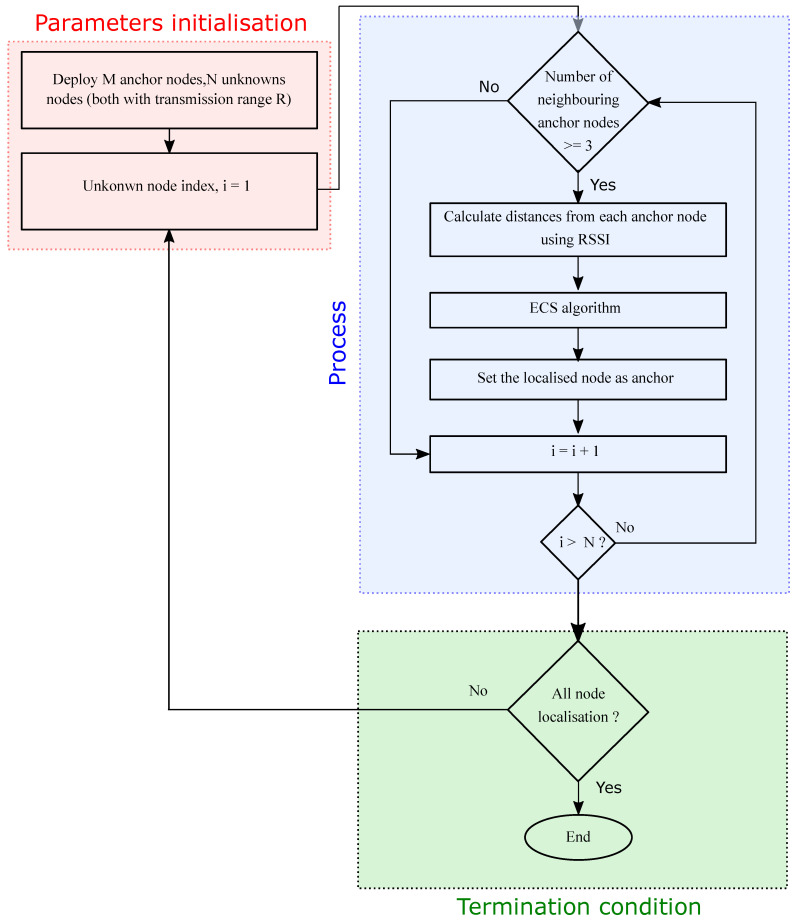
Node localisation process in WSNs.

Node localisation in WSNs is done to estimate the coordinates of *N* unknown nodes using the coordinates of the known *M* anchor nodes. The node localisation process consists of the following steps (also illustrated in Figure 3).

1. Deploy a finite *M* number of anchor nodes and *N* number of unknown nodes; each node is assumed to have a homogeneous transmission range equal to *R*.
2. When the *i*-th unknown node has three or more anchor nodes within its transmission range, only then is it considered localisable. Afterwards, the distances of each unknown node from its neighbouring anchors are calculated using Equations (3) and (4).
3. Establish the objective function using Equation (5).
4. Each unknown node attempts to localise itself by running the ECS algorithm independently.
5. ECS algorithm estimates the optimal positions for all the localisable nodes by minimising the ranging error. After getting localised, each unknown node starts acting as an anchor node and helps other localisable nodes get localised, indicating the increase in the number of anchor nodes as the iteration count progresses.
6. Repeat steps 2–5 until all the localisable nodes become localised.
7. The performance of the node localisation process is analysed in terms of ALE and Localisation Success Ratio (LSR). These parameters are discussed in the simulation analysis in Section 3.1.

**Algorithm 1:**ECS algorithm

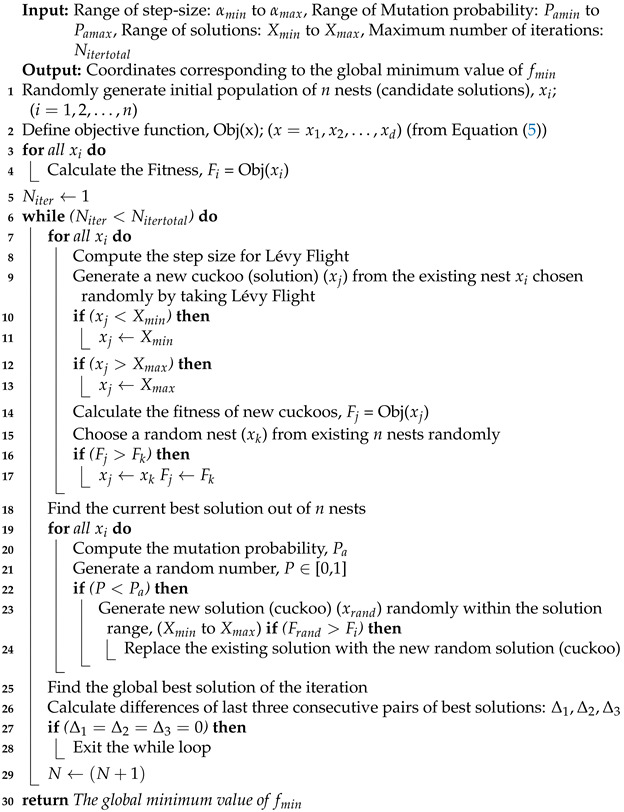



## 3. Simulation Experiment

In this section, we discuss the simulation environment for the performance evaluation of the proposed ECS algorithm. Cheng et al. [37] have compared the performance of modified CS algorithm with GA, PSO and other traditional optimisation algorithms. They found that the modified CS algorithm outperforms other algorithms in terms of localisation accuracy and computation complexity with respect to node localisation. Inspired by this, we have compared the performance of modified CS and the proposed ECS algorithm to accomplish the same task under the same setup.

### 3.1. Simulation Analysis Parameters

The following parameters are used to study and analyse the performance of the proposed ECS algorithm.

1. Average Localisation Error (ALE)
  ALE indicates how effectively the localisation technique estimates the position of the unknown nodes of the network. ALE is defined as the mean of the Euclidean distance between the estimated node location and the actual unknown node location of the sensor nodes, as shown by Equation (7)
  (7) $$ALE=\frac{\sum_{i=1}^{N_{L}}\sqrt{{\left(X_{i}-x_{i}\right)}^{2}+{\left(Y_{i}-y_{i}\right)}^{2}}}{N_{L}}$$
  where $N_{L}$, $\left(x_{i},y_{i}\right)$ and $\left(X_{i},Y_{i}\right)$ represent the number of localised nodes, estimated node position and the actual position of the unknown node, respectively. It is impossible to eliminate ALE because of the non-zero ranging error between two nodes, which manipulates the distance calculation of the unknown node. The ranging error diminishes the probability of the anchor nodes being placed at the exact position of unknown nodes. Therefore, ALE should be as small as possible. Here, a lower value of the ALE represents higher accuracy of the localisation algorithm.
2. Localisation Success Ratio (LSR)
  LSR is defined as the ratio of the number of localised nodes to the total number of unknown nodes in the network and can be calculated by Equation (8):
  (8) $$LSR=\frac{\mathrm{Number}\;\mathrm{of}\;\mathrm{unknown}\;\mathrm{nodes}\;\mathrm{localised}}{\mathrm{Total}\;\mathrm{number}\;\mathrm{of}\;\mathrm{unknown}\;\mathrm{nodes}}\times 100\%$$
  A higher value of LSR represents that the localisation algorithm performs better.

### 3.2. Simulation Setup

To perform the simulations, we have considered a 2-dimensional rectangular-shaped region where nodes are distributed randomly and uniformly. Anchor nodes are assumed to be free from any localisation error. The various simulations parameters, along with their description and values, are provided in Table 1.

## 4. Simulation Results and Analysis

Here, we provide the simulation outcomes of our proposed ECS algorithm and compare them with the modified CS algorithm results. Further, we also discuss the impact of the ES criterion, the number of iterations and anchor node density in the network on the network parameters ALE and LSR.

### 4.1. The Impact of the ES Criterion

As discussed earlier, the modified CS algorithm [37] has a faster convergence rate as compared to GA, PSO and SA. However, the modified CS algorithm still searches for the best solution in the search space until the last iteration (set to 100 in our case) for each unknown node. This process consumes a lot of time and resources of the sensor nodes. In contrast, the proposed ECS algorithm converges quickly and leaves the search loop to further localise the other nodes due to the ES criterion’s implementation in the search.

**Figure 4:**
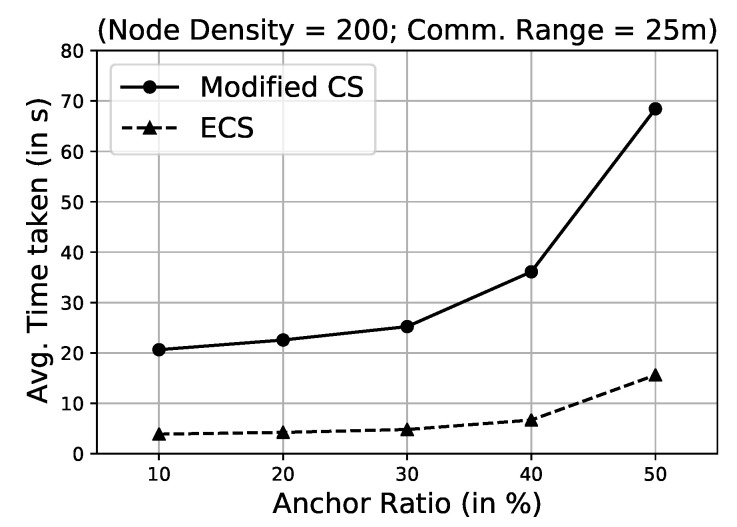
Comparison of modified Cuckoo Search (CS) algorithm and the proposed Enhanced Cuckoo Search (ECS) algorithm based on average time taken to localise one localisable node.

To compare the modified CS algorithm with the proposed ECS algorithm, we have first created a comparison environment where we kept all the parameters constant, except the method used to solve the problem. We consider a 100 m × 100 m monitoring region where 100 sensor nodes are spread randomly and uniformly. Each sensor node was assumed to have a homogeneous transmission range of 25 m. We calculated the average time taken by modified CS and ECS to localise a single node by varying the anchor ratio from 10% to 50% (see Figure 4). We found that the average time taken by an unknown node to localise itself using the modified CS algorithm is almost four times the average time taken using the proposed ECS algorithm. There is an approximately 80% decrease in the average time taken to localise a localisable node when the proposed ECS algorithm is applied as compared to the modified CS algorithm. On an average, each node is localised within 10 s. As the anchor ratio is increased from 10% to 50%, the number of neighbouring anchor nodes increases for an unknown node, which increases the computational time for realising the position of the unknown node. In the modified CS algorithm, the average time taken to localise an unknown node is high. In contrast, the proposed ECS algorithm localises the unknown nodes in a reasonable time that is feasible for practical applications.

### 4.2. Comparison of Modified CS and ECS Algorithm

**Figure 5:**
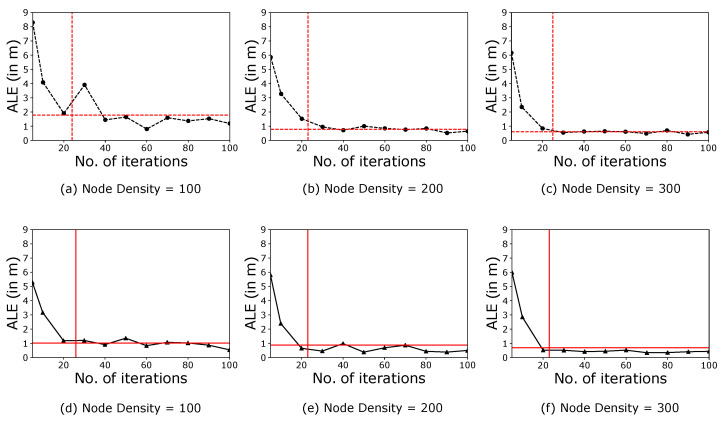
Variation of ALE with respect to the number of iterations for a transmission range of 15 m over a monitoring area of 100 m × 100 m.

**Figure 6:**
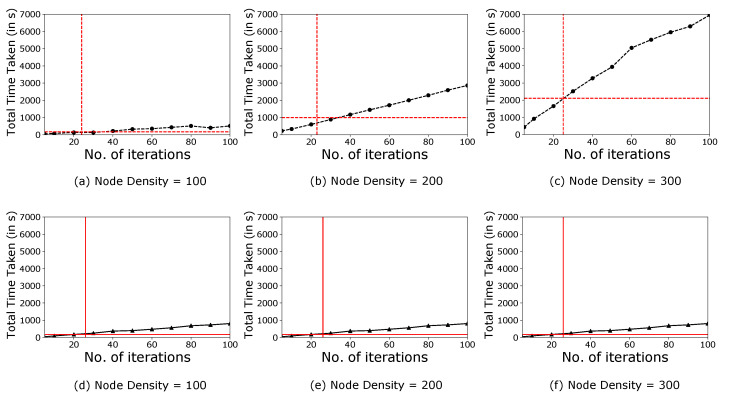
Variation of total time taken with respect to the number of iterations for a transmission range of 15 m over a monitoring area of 100 m × 100 m.

We have obtained the outcomes of the proposed ECS algorithm and compared them with the results obtained from the modified CS algorithm. In doing so, first, we have plotted the obtained ALE for the number of iterations (see Figure 5). This plot enables the comparison of both the algorithms based on their accuracy to localise all the localisable nodes. Afterwards, we have plotted the time taken by the system model to localise the nodes for the number of iterations (see Figure 6). This plot enables the comparison of both the algorithms based on their speed.

In Figure 5a–c, the dotted black line represents the variation of ALE with 15% anchor ratio for modified CS algorithm. Similarly, the solid black line represents the variation of ALE with a 30% anchor ratio for the modified CS algorithm (see Figure 5d–f). The intersection of the dotted red line represents the iteration number where the ECS algorithm exits the search process on the x-axis and the corresponding ALE on the y-axis for 15% anchor ratio (see Figure 5a–c). Similarly, the intersection of solid red corresponds to 30% anchor ratio (Figure 5d–f). When the search process starts for the node localisation, we found that both the algorithms begin with a random population of candidate solutions having spontaneous ALE, which is generally large due to the random nature of the estimated positions. As the number of iterations increase, the coordinates with the best fitness value in the iteration improve significantly. However, the ECS algorithm completes the node localisation task in a fewer number of iterations. Furthermore, we found that the ECS algorithm performs promisingly for 15% anchor ratio, and hence it is practically feasible to use ECS compared to modified CS. Figure 6 follows the same representation as that of Figure 5. On comparing both the algorithms in terms of time taken, we found that the ECS algorithm performs way better than the modified CS algorithm for both 15% and 30% anchor ratio. Therefore, the proposed ECS algorithm is computationally efficient.

### 4.3. Dependency of ECS Algorithm on Anchor Density

The anchor density parameter is essential in a way that it influences both the localisation performance and cost of the network. Therefore, a good WSN should need as fewer as possible anchor nodes at the time of deployment. In our simulations, the anchor density is varied from 10% to 50% of all the sensor motes. In this observation, the transmission range of each node is fixed at 25 m.

**Figure 7:**
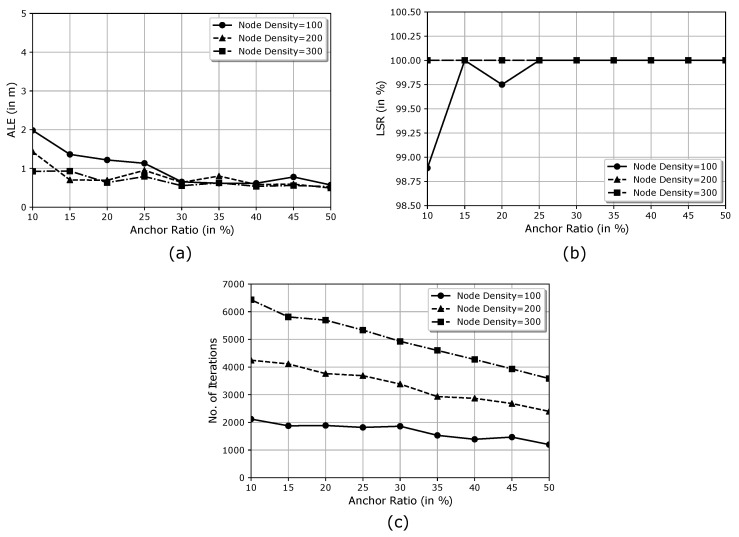
Effect of anchor ratio variation on the performance of the proposed ECS algorithm. (**a**) The effect of anchor ratio variation on ALE. (**b**) The effect of anchor ratio variation on LSR. (**c**) The effect of anchor ratio variation on the performance of the proposed ECS algorithm.

- ALE
  Figure 7a displays the variation effect of the anchor ratio on ALE. We found that the ALE decreases with an increase in the anchor ratio from 10% to 50%. However, the value of ALE does not vary much with the anchor ratio, as it remains between 0.5 m and 2 m. Furthermore, the value of ALE decreases as the node density increases. This decrease owes to the network connectivity getting better with the increase in population density of the nodes.
- LSR
  Figure 7b shows the variation of the anchor density on LSR for different node densities in the network. We found that the ECS algorithm can localise all the localisable nodes with an anchor ratio of just 30% and node density of 100 nodes. As the node density increases to 200 nodes, the ECS algorithm can localise all the unknown nodes present in the network with an anchor ratio of a mere 10%. A similar trend is observed when the node density is increased to 300. In Figure 7b, the observation points overlap each other for node density 200 and 300. Therefore, we can reduce the network cost using the ECS algorithm and make it less dependent on the GPS-assisted nodes.
- Number of Iterations ($N_{iter}$)
  Figure 7c demonstrates that the number of iterations required to localise all the possibly localisable nodes decreases with increasing anchor ratio. This trend is observed because the number of unknown nodes to be localised reduces as the anchor ratio increases from 10% to 50%. As we increase the node density, the required number of iteration also increases.

The ECS algorithm performance does not depend significantly on the number of initially deployed anchor nodes (see Figure 7a). It requires a minimum of three anchor nodes in the communication range of an unknown node to find the best solution. Furthermore, the time required to localise every node increases with the increase in the anchor ratio (Figure 4) because of the following three reasons: first, the mathematical calculations required to estimate the localisable nodes’ increase with the increase in the anchor ratio; second, the presence of an increasingly greater number of GPS-assisted nodes at the time of deployment; and last, increased chances of anchor nodes being in the communication range of unknown nodes.

## 5. Discussion

The proposed ECS algorithm is a search algorithm that can be used to solve a wide range of optimisation problems. In the WSNs domain, ECS can find applications in node localisation, data aggregation, determining optimal coverage and energy-efficient clustering and routing [41]. In this paper, we have shown that how ECS can be applied to solve the node localisation issue in WSNs. The results are promising. Recently, Singh et al. [42] proposed an approach that uses a hybrid meta-heuristic algorithm using Improved Genetic Algorithm and Binary Ant Colony Algorithm (IGA-BACA) for optimal coverage with minimum redundant information. Still, the process has a high computational cost. Here, we can use ECS to reduce the computational cost associated with the optimal coverage problem in WSNs. More recently, various studies have been reported using conventional CS algorithms for Cluster Head (CH) selection [43] whose results concerning the time-complexity aspect can be improved further using the proposed ECS algorithm. Furthermore, the proposed ECS algorithm can be extended further to build an energy consumption model which can keep track of the consumed energy during the node localisation process.

## 6. Conclusions

The proposed ECS algorithm shows promising results for the node localisation in the domain of the WSNs. On comparing the time complexity of the proposed ECS algorithm with that of the modified CS algorithm, the former is proved to provide the results faster than the latter. The implementation of the ES criterion causes this to exit the search loop earlier than deciding if the global optimum solution is reached. ECS algorithm performs well even when the values of node density, anchor ratio, and transmission range of each sensor are kept considerably low. It achieved accurate results (low ALE) with high LSR in such network conditions.

This study is a step towards time-efficient optimisation and can be applied in diverse applications for natural and nearly real optimisation problems.

## Figures and Tables

**Table 1:** Simulation parameters.

Simulation Parameter	Description	Value
Deployment Area (in m${}^{2}$)	The application area of the WSN where all the sensor nodes are deployed	100 × 100
Node Density	It is defined as the total number of sensor nodes in the given test area	100, 200, 300
Anchor Ratio (in %)	Percentage of anchor nodes out of the total nodes in the network	10, 20, 30, 40, 50
Communication Range (in m)	The distance up to which a sensor node can communicate	10, 20, 30, 40, 50
α	Step size	0.9–1.0
$P_{a}$	Mutation probability	0.05–0.25
$N_{nest}$	Number of candidate solutions	25
$N_{itertotal}$	Maximum number of iterations allowed to localise each unknown node	100

## Data Availability

The code can be downloaded from https://github.com/vkotiyal/thesis-ecs-codes (accessed on 20 May 2021).
